# A meta-analysis of obstetric and neonatal outcomes in patients after treatment of hysteroscopic adhesiolysis

**DOI:** 10.3389/fendo.2023.1126740

**Published:** 2023-03-24

**Authors:** Xu Wenzhi, Xu Xin, Zhou Ping, Wu Hanglin, Lin Xiaona

**Affiliations:** ^1^ Department of Obstetrics and Gynecology, Sir Run Run Shaw Hospital, School of Medicine, Zhejiang University, Hangzhou, China; ^2^ Key laboratory of Reproductive Dysfunction Management of Zhejiang Province, School of Medicine, Zhejiang University, Hangzhou, China; ^3^ Department of Obstetrics and Gynecology, Hangzhou Women’s Hospital, Hangzhou, China

**Keywords:** intrauterine adhesion, hysteroscopic adhesiolysis, obstetric outcomes, placentarelated disorders, postpartum hemorrhage

## Abstract

**Introduction:**

Hysteroscopic adhesiolysis is widely performed in women with intrauterine adhesions. Small observational studies have reported the obstetric and neonatal outcomes, but studies with larger sample sizes are few. The aim of this study is to evaluate the obstetric and neonatal outcomes in women after hysteroscopic adhesiolysis.

**Methods:**

We conducted a literature search in July 2022 using the PubMed, Embase, the Cochrane Library, and Web of Science databases, and finally, 32 studies (N = 3812) were included. We did a meta-analysis to estimate the prevalence of placenta-related disorders, including placenta previa, placental abruption, placenta accreta, placenta increta, and retained placenta. We also included other obstetric and neonatal outcomes like postpartum hemorrhage, ectopic pregnancy, oligohydramnios, gestational hypertension, gestational diabetes mellitus, and intrauterine growth restriction. The results were presented as odds ratios (ORs) with 95% confidence intervals (CIs) in studies with a control group, but otherwise as prevalence (%) with 95% confidence intervals (CIs).

**Results:**

The overall pregnancy and live birth rates were 58.97% and 45.56%, respectively. The prevalence of placenta previa differed in pregnant women who underwent hysteroscopic adhesiolysis compared with those who did not (OR, 3.27; 95% CI, 1.28-8.36). In studies without a comparative group, the pooled rate of placenta accreta was 7% (95% CI, 4-11) in 20 studies; placenta increta was 1% (95% CI, 0-4) in 5 studies; a retained placenta was 11% (95% CI, 5-24) in 5 studies; postpartum hemorrhage was 12% (95% CI, 8-18) in 12 studies; ectopic pregnancy was 1% (95% CI, 0-2) in 13 studies; oligohydramnios was 3% (95% CI, 1-6) in 3 studies; intrauterine growth restriction was 3% (95% CI, 1-8) in 3 studies; gestational hypertension was 5% (95% CI, 2-11) in 4 studies; and diabetes mellitus was 4% (95% CI, 2-7) in 3 studies.

**Discussion:**

Due to the paucity of good quality comparative data, the question of whether there is an increased prevalence of obstetric and neonatal complications in women after hysteroscopic adhesiolysis compared with the general population remains unanswered. The findings from this review will provide a basis for more well-designed studies in the future.

**Systematic review registration:**

https://www.crd.york.ac.uk/PROSPERO/display_record.php?RecordID=364021, identifier [CRD42022364021].

## 1 Introduction

The etiology of intrauterine adhesions (IUA) was first reported by Joseph Asherman in 1948 (1). Uterine adhesions are strips of fibrous tissue within the uterine cavity, which can form a thin band of adhesions in mild cases or lead to complete occlusion of the uterine cavity in severe cases. Common causes of uterine adhesions include miscarriage, invasive intrauterine operations, inflammation or infection, and uterine compression stitches. The prevalence of uterine adhesions in women after spontaneous abortion has been reported in the literature to be up to 19.1% (2). Some patients with mild uterine adhesions may have no clinical manifestations, whereas patients with Asherman syndrome mainly present with secondary amenorrhea or hypomenorrhea. A review reported the following manifestations of abnormal uterine bleeding in patients with uterine adhesions: amenorrhea (37%), scanty bleeding (31%), normal menstruation (5%), and excessive menstruation (1%) (3). Additionally, about 7% to 40% of patients with uterine adhesions exhibit infertility (3), which may be due to damage to the endometrium that prevents embryo implantation (4).

Currently, hysteroscopic adhesiolysis remains the standard treatment for intrauterine adhesions. This can be supplemented by postoperative physical isolation or hormonal therapy to prevent the formation of adhesions. The current data on reproductive outcomes after IUA treatment are mainly from small observational studies. In a systematic evaluation, pregnancy rates of 40% to 80% and live birth rates of 30% to 70% were reported after treatment of uterine adhesions (5). The placenta accreta spectrum was seen in nearly 10% of patients with postoperative pregnancies (6).

There are few systematic reviews of the incidence of obstetric complications after hysteroscopic adhesiolysis. In 2019, Guo et al. analyzed 54 publications and reported the integrated incidences of ectopic pregnancy, pregnancy loss, placenta previa, placenta abruption, postpartum hemorrhage, placenta accreta syndrome, premature rupture of membranes, cervical insufficiency, intrauterine growth restriction, and preterm delivery. However, a detailed distinction was not made between the placenta accreta spectrum, the analysis had no control group, and the final incidence of integration obtained was compared with the general population, which created a large bias (7). In 2021, Hooker reviewed 5 papers that analyzed the obstetric outcomes of mild intrauterine adhesions, but the associated obstetric complications were not mentioned (30). Our systematic evaluation includes obstetric complications and adds studies published in the last 3 years, thus providing updated data on the incidence of obstetric complications in patients with pregnancies after hysteroscopic adhesiolysis. We assess the need to increase pregnancy screening in these patients in clinical practice.

## 2 Materials and methods

The Preferred Reporting Items for Systematic Reviews and Meta-Analyses (PRISMA) standards were followed for conducting this systematic review (8). Institutional Review Board approval was not obtained because all data were taken from previously published data.

### 2.1 Search strategy

In July 2022, the following electronic databases were extensively searched for scientific literature: PubMed, Embase, Cochrane Library, and Web of Science. The search terms used were “intrauterine adhesions”, “hysteroscopy”, “obstetric outcomes”, “pregnancy rate”, “miscarriage”, “placenta previa”, “placental abruption”, “placenta accreta”, “placenta increta”, “retained placenta”, “postpartum hemorrhage”, “oligohydramnios”, “ectopic pregnancy”, “gestational hypertension”, “gestational diabetes mellitus” (GDM), “intrauterine growth restriction”, and their variants. These were restricted to the title, abstract, and keywords (see **Supplementary Materials** for a detailed search strategy). The research protocol (CRD42022364021) has been desposited into the International Prospective Register of Systematic Reviews (PROSPERO) database.

### 2.2 Outcome measures

We included placenta-related disorders including placenta previa, placenta accreta, placenta increta, placental abruption, and retained placenta together with postpartum hemorrhage as the primary outcomes. Placenta previa is defined as the placenta completely or partially covering the internal cervical os (9). Placenta accreta is defined as an attachment of the placenta to the myometrium without intervening decidua, and placenta increta is an invasion of the trophoblast into the myometrium (10). The classic definition of placental abruption is a premature separation of the placenta before delivery (11). Retained placenta after vaginal delivery is diagnosed when a placenta does not naturally deliver within a predetermined window of time, typically between 18 and 60 minutes (33).

Associated secondary outcomes included ectopic pregnancy, intrauterine growth restriction, oligohydramnios, gestational hypertension, and gestational diabetes mellitus. Ectopic pregnancy refers to an extrauterine pregnancy and the fallopian tube is the most common site (31). Intrauterine growth restriction is the failure of a fetus to achieve its intrinsic growth potential, often characterized as low weight, length, or head circumference (12). Oligohydramnios is characterized by an amniotic fluid volume that is less than the minimum anticipated for the corresponding gestational age. This condition is best identified by ultrasound and includes amniotic fluid index (AFI) values of less than 5 cm and single deepest pockets (SDP) of less than 2 cm (13). Gestational hypertension is defined as having a blood pressure of ≥ 140/90 mmHg at least two times more than 4 hours apart after 20 weeks of gestation (32). GDM refers to any degree of glucose intolerance that was first identified during pregnancy (14). Where definitions differed, we accepted the definition provided by the authors of the original study.

### 2.3 Paper selection and eligibility

We included studies that reported on obstetric and neonatal outcomes in all women diagnosed with intrauterine adhesions after hysteroscopic adhesiolysis. All retrospective and prospective studies with 10 or more cases were included. We retrieved studies with both a single arm and a comparison arm. Case reports, reviews, comments, letters, and conference abstracts were excluded. Only studies reported in English were included.

Two authors independently selected the studies in a two-stage process. All titles and abstracts were first individually reviewed, and the agreement for possible relevance was accomplished by consensus. Second, an examination of the full manuscript was carried out to ascertain eligibility. Significant data about the study characteristics and relevant outcomes were extracted independently. When additional information was needed, we contacted the study author by email.

### 2.4 Data extraction and statistical analysis

Characteristics retrieved from all studies included author, country, year of publication, design, number of subjects, mean age of sample population, classification ofIUA, mean duration of follow-up, and outcomes reported. The collected outcome details included the event number and total number. All the data analysis and the graphical renderings were carried out using R version 4.2.1. We calculated the proportion with a 95% confidence interval (CI) of the different outcomes for each study. We assessed statistical heterogeneity by visual inspection of the forest plot, applied the Q test for heterogeneity, and calculated the *I^2^* statistic. A fixed effects model was used if an *I^2^* ranged from 0% to 25%; otherwise, a random effects model was used. When the event number had a value of 0, double-arcsine conversion is used. If 10 or more studies were under consideration, we drew a funnel diagram to assess publication bias.

## 3 Results

### 3.1 Search results

**Figure 1:**
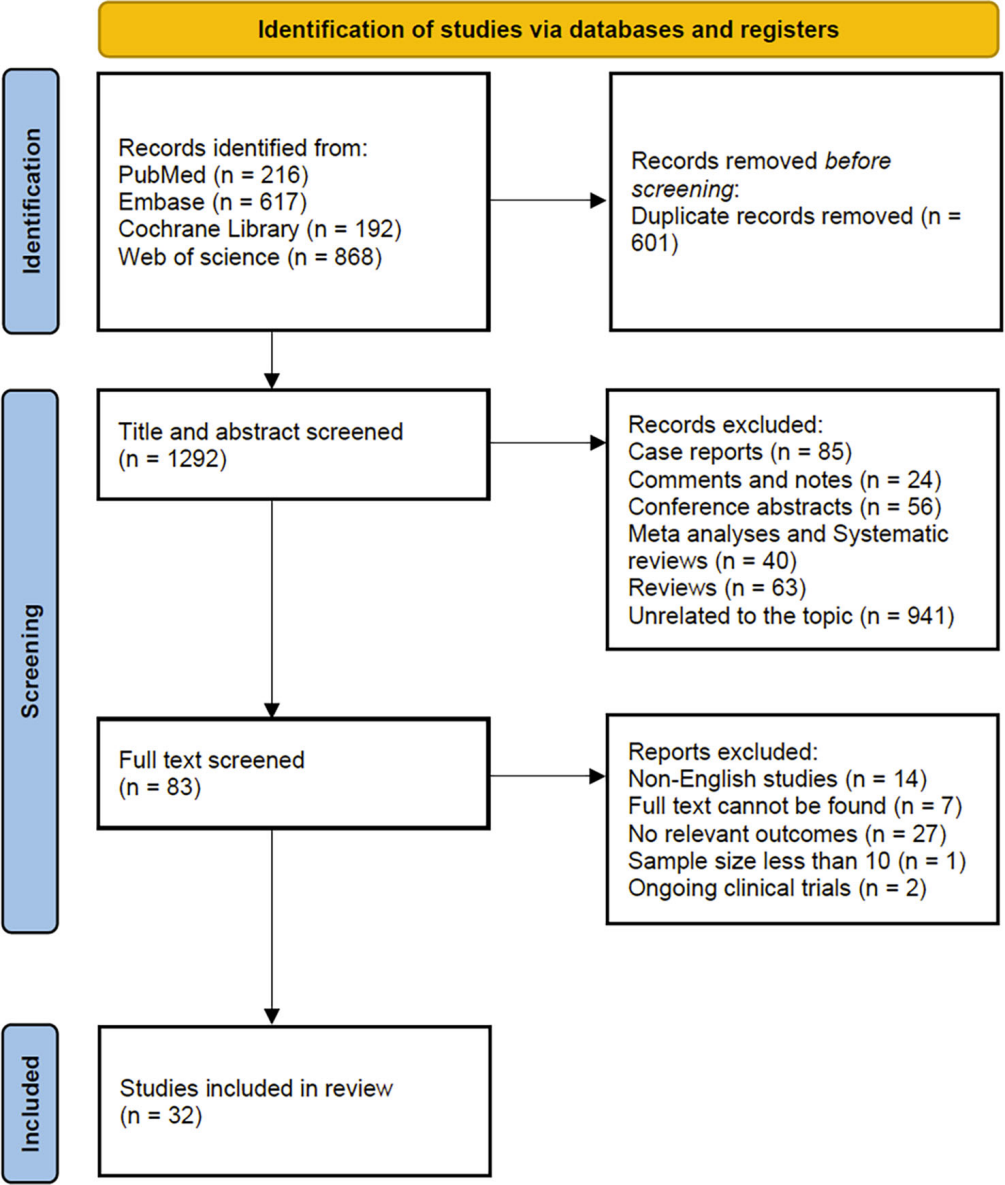
Flow chart of the study.

As shown in the flow chart (**Figure 1**), 1893 studies (PubMed = 216; Embase = 617; Cochrane Library = 192, Web of Science = 868) were identified. After removing duplicates, 1292 studies remained, and 941 studies unrelated to the topic were removed. After evaluating titles and abstracts, 83 studies remained. After a full-text assessment, 51 of these studies were excluded due to different reasons (e.g., non-English studies, the full text could not be found, no relevant outcomes, sample size less than 10, and ongoing clinical trials). Finally, 32 studies were included in the analysis.

### 3.2 Overview of included studies

Three publications—two retrospective cohort studies and one case-control study—reported obstetric outcomes in patients after hysteroscopic adhesiolysis and compared them with patients without IUA. The remaining 29 studies had no comparison group. A summary of the included studies is presented in **Supplementary Table 1**.

### 3.3 Risk of bias

**Figure 2:**
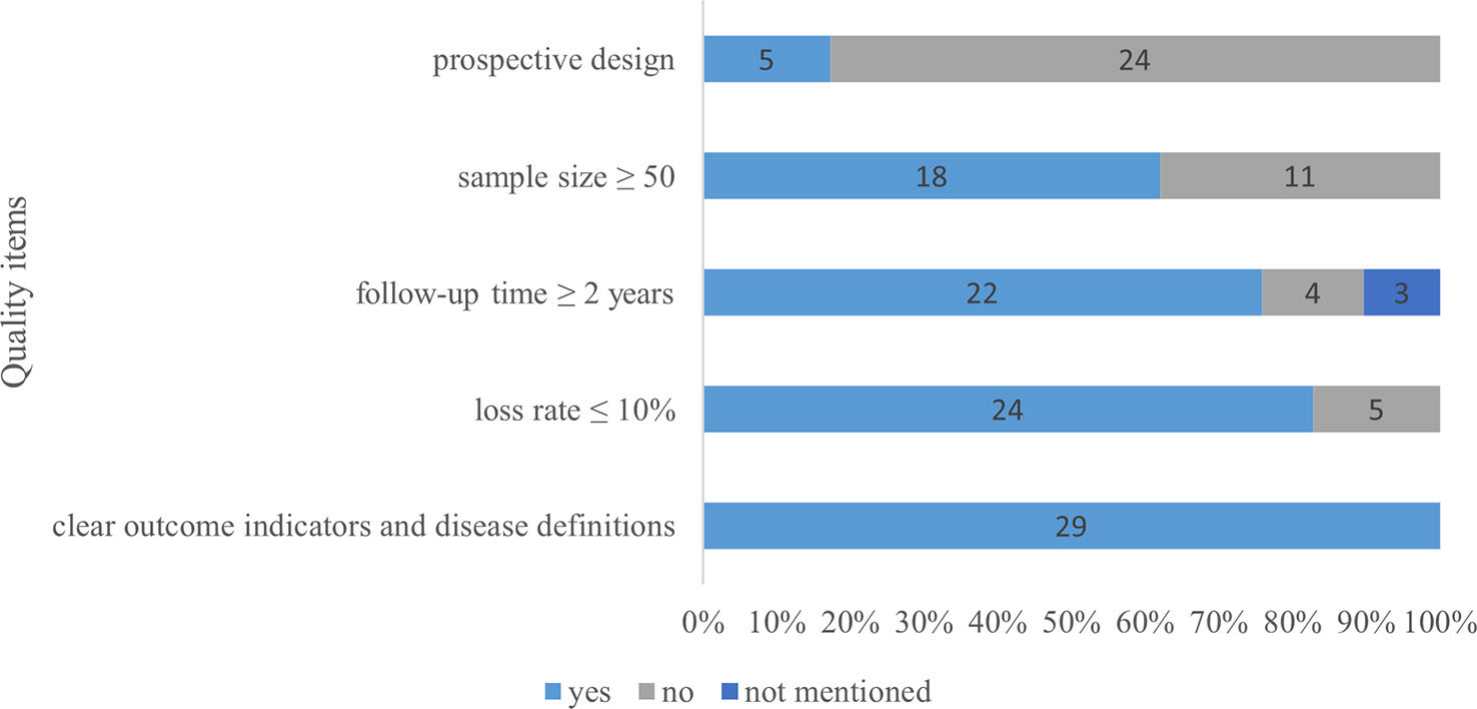
The quality of the papers.

Two authors independently assessed the risk of bias using the Newcastle-Ottawa Scale in the three studies with a comparison group. The results are summarized in **Table 1**. All three studies scored 8 points. None of the three studies indicated whether the control group came from the same population as the exposure group or case series. Other included studies were evaluated in terms of clear outcome indicators and disease definitions, such as retrospective design, loss rate ≤ 10%, follow-up time ≥ 2 years, prospective study, and sample size ≥ 50. The results are shown in **Figure 2**.

**Table 1:** Risk of bias assessment using the Newcastle-Ottawa Scale (for studies with a comparator group).

Author	Year	Selection	Comparability	Exposure
luping Zhang	2020	1+0+1+1	2	1+1+1
yuqing Wang	2020	1+0+1+1	2	1+1+1
Saeed Baradwan	2018	1+1+0+1	2	1+1+1

### 3.4 Pregnancy rate and live birth rate

The pregnancy rate is defined as the success rate of getting pregnant. The live birth rate is the percentage of children born who showed any signs of life. The 32 studies analyzed reported 2251 pregnancies out of 3817 women resulting both naturally and from assisted reproduction. Thus, the pregnancy rate was 58.97%. Live births were reported in 23 studies with 1370 live births occurring in 3007 women, thus the live birth rate was 45.56%.

### 3.5 Primary outcomes

**Figure 3:**
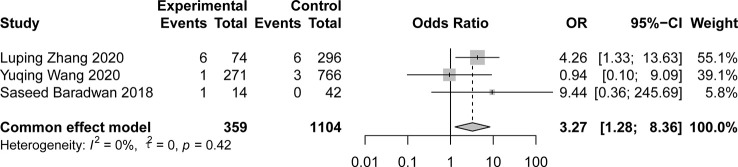
Pooled fixed effects estimate of placenta previa.

**Figure 4:**
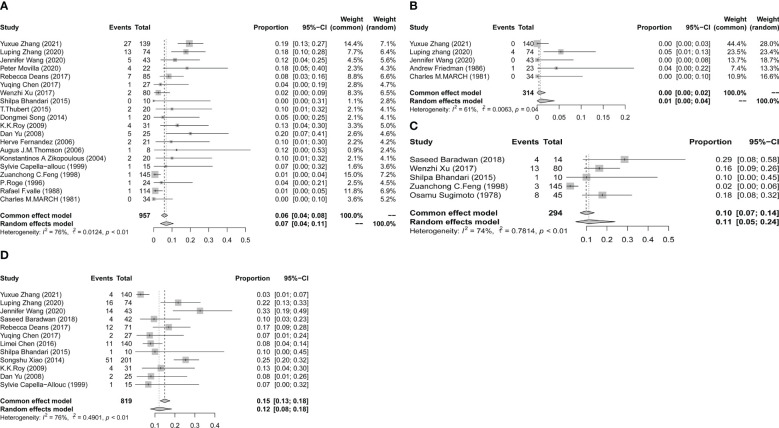
Forest plot diagrams of placenta accreta **(A)**, placenta increta **(B)**, retained placenta **(C)**, and postpartum hemorrhage **(D)**. (total number: number having live births).

Three out of the 32 studies had a control group with a sample size three times larger than the exposed group. These studies showed comparable results in both arms in terms of age, BMI, and pregnancies at baseline. All three studies described the incidence of placenta previa. **Figure 3** shows that a history of hysteroscopic adhesiolysis in pregnant women resulted in a significant increase in the incidence of placenta previa compared with unexposed pregnant women (OR, 3.27; 95% CI, 1.28-8.36; *I^2^* = 0%). Only two studies reported the prevalence of placental abruption after hysteroscopic adhesiolysis, which was 0 cases in 117 women. For other outcomes, we conducted a meta-analysis of individual rates as summarized in **Figure 4**. Placenta accreta occurred in 80 of the 957 women, resulting in a pooled proportion of 7% (95% CI, 4-11; *I^2^* = 76%; *P*<0.01). Placenta increta occurred in 5 of the 314 women, resulting in a pooled proportion of 1% (95% CI, 0-4; *I^2^* = 61%; *P* = 0.04). Retained placenta occurred in 29 of the 294 women, resulting in a pooled proportion of 11% (95% CI, 5-24; *I^2^* = 74%; *P*<0.01). Postpartum hemorrhage occurred in 122 of the 819 women, resulting in a pooled proportion of 12% (95% CI, 8-18; *I^2^* = 76%; *P*<0.01). To evaluate publication bias, we drew funnel plots for outcomes that have been discussed in more than 10 articles. The results are displayed in **Supplementary Figure 2**. The results of Egger’s experiment showed that there is no significant publication bias for placenta accreta (*P* = 0.097) and postpartum hemorrhage (*P* = 0.968).

**Table 2** shows that compared with the general population, the prevalence of placenta accreta was increased, whereas no difference in the incidence of placenta increta, retained placenta, and postpartum hemorrhage was observed.

**Table 2:** Prevalence of obstetric and neonatal outcomes after hysteroscopic adhesiolysis in IUA women with the rates in the general population.

Obstetric and neonatal complications	No. of cases	Reported articles	Pooled prevalence (%, 95%CI) in IUA population	*I^2^*	General population (%)
Placenta accreta	80/957	20	7 (4-11)	76%	0.04-0.9(15)
Placenta increta	5/314	5	1 (0-4)	61%	0.01-0.16(9)
Retained placenta	29/294	5	11 (5-24)	74%	0.5-3(16)
Postpartum hemorrhage	122/819	12	13 (8-18)	76%	3-8(17)
Ectopic pregnancy	32/1531	13	1 (0-2)	26%	1-2(18)
Oligohydramnios	6/263	3	3 (1-8)	69%	1-5(19)
Gestational hypertension	24/528	3	5 (2-11)	82%	4-25(20)
Gestational diabetes mellitus	17/454	4	4 (2-7)	66%	7.6-9.2(21)
Intrauterine growth restriction	7/258	3	3 (1-8)	69%	3-7(22)

CI, confidence interval.

### 3.6 Secondary outcomes

**Supplementary Figure 1** and **Table 2** show no significant difference in the prevalence of ectopic pregnancy, oligohydramnios, gestational hypertension, gestational diabetes mellitus, and intrauterine growth restriction in pregnant women after hysteroscopic adhesiolysis compared with the general population.

## 4 Discussion

The overall conception rate for patients after hysteroscopic adhesiolysis was 58.97% and the live birth rate was 45.56%. This coincides with existing studies that report pregnancy rates between 25% and 76% and term delivery rates between 25% and 79.7% (23, 24). These rates are lower compared with the general population likely because the surgical operation can lead to more severe adhesions.

Our study found an increased incidence of placenta previa in the population that had hysteroscopic adhesiolysis compared with the general population (OR, 3.27). The mechanism by which placenta previa occurs is not fully understood. One theory suggests that the placenta itself does not move; instead, the placental rim atrophies in areas with inadequate vascular supply, but migrates and continues to grow in more vascular sites (25). We speculate that uterine adhesions or resultant surgical procedures cause the poor decidualization of the vasculature in the upper segment of the uterine cavity, making it unsuitable for embryo implantation.

In 2019, Guo et al. published a meta-analysis on the outcomes of patients with IUA who underwent adhesiolysis that focused on pregnancy outcomes and obstetric complications. In contrast, our systematic review only included literature that reported obstetric complications and not pregnancy outcomes. The placenta accreta spectrum, formerly known as morbidly adherent placenta, comprises placenta increta, placenta percreta, and placenta accreta. It refers to a range of pathologic adhesions of the placenta (10). We subdivided the placenta accreta spectrum into placenta accreta, placenta increta, and retained placenta for separate analyses. The analysis by Guo et al. on the placenta accreta spectrum included 23 papers, and the pooled prevalence was 10.1% (95% CI, 8.6-11.8), whereas we obtained a prevalence of 7% from the results of 20 papers. Moreover, 5 papers reported a placenta increta prevalence, with a pooled prevalence of 1%, 5 papers reported a retained placenta prevalence, and the pooled prevalence was 11%. Overall, our placental disease prevalence was slightly reduced compared with that reported by Guo et al. Similarly, we found a decrease in the prevalence of ectopic pregnancy (1% vs 4.2%) and intrauterine growth restriction (3% vs 8.4%) compared with those from Guo et al. Possible reasons for these differences are as follows: first, we included literature published in the last 3 years to acquire more accurate results; second, hysteroscopic equipment has improved over the years. The main instruments used in the treatment of IUA include sharp or blunt scissors (23), monopolar (26), or bipolar diathermy (27). More physicians have realized that careful dissection is needed with instruments that produce heat to avoid destroying the endometrium. As a result, in recent years, patients have had relatively less postoperative endometrial damage and severe complications. Furthermore, there is an increased use of estrogen (5), intrauterine devices (28), or Foley catheters (29) after an operation, which helps to prevent the recurrence of uterine adhesions. So, IUA prognosis and pregnancy outcomes are improving. This is in line with the reported increase in postoperative pregnancy rates with modern advances by Guo et al.

According to our study, the prevalence of postpartum hemorrhage in the target population was 8% to 18%. This may be associated with an increased prevalence of placenta-related disorders and the retention of placental membranes.

Most obstetric complications are comparable to those in the general population. The etiology of gestational diabetes mellitus and gestational hypertension is extremely complex, encompassing both genetic and environmental factors. Therefore, more detailed case information and subgroup analysis are needed to further explore the relationship between hysteroscopic adhesiolysis and their prevalence. These findings suggest that we should intensify monitoring this population during all stages of pregnancy.

## 5 Limitations

This study has several limitations: (1) Only three of the included studies have control groups, and the outcomes of these studies are not uniform and comprehensive; (2) The design types of the remaining 29 studies are not uniform and included case series, case-control studies, cohort studies, etc. Therefore, we could only set some quality evaluation items, and the quality of the included literature cannot be comprehensively estimated; (3) Age or assisted reproduction are confounding factors of obstetric complications, but some literature did not make stratified statistics on the above factors, so confounding bias cannot be evaluated at present; (4) Large publication bias exists in the analysis of ectopic pregnancy.

## 6 Conclusions

Due to the paucity of good quality comparative data, the question of whether there is an increased prevalence of obstetric and neonatal complications in patients after hysteroscopic remains unanswered. However, with the help of the summary data provided in this study, more well-designed studies can be carried out in the future.

## Data availability statement

The original contributions presented in the study are included in the article/**Supplementary Material**. Further inquiries can be directed to the corresponding author.

## Author contributions

LX was responsible for designing the study. XW contributed to selecting papers, carrying out the meta analyses and writing up; XX participated in selecting papers, data abstraction, carrying out the meta analyses and contributed to writing up; ZP and WH provided knowledge and participated in the quality control of the study. All authors contributed to the article and approved the submitted version.

## Supplementary material

Supplementary Figure 1 Forest plot diagrams of ectopic pregnancy **(A)**, oligohydramnios **(B)**, intrauterine growth restriction **(C)**, gestational hypertension **(D)**, and gestational diabetes mellitus **(E)**.

Supplementary Figure 2 The funnel plots of placenta accreta **(A)**, postpartum hemorrhage **(B)** and ectopic pregnancy **(C)** which have more than 10 studies reported.

## References

[1] Asherman JG . Amenorrhoea traumatica (Atretica). BJOG Int J Obstet. Gynaecol. (1948) 55:23–30. doi: 10.1111/j.1471-0528.1948.tb07045.x 18902559

[2] Hooker AB Lemmers M Thurkow AL Heymans MW Opmeer BC Brolmann HAM . Systematic review and meta-analysis of intrauterine adhesions after miscarriage: prevalence, risk factors and long-term reproductive outcome. Hum Reprod Update (2014) 20:262–78. doi: 10.1093/humupd/dmt045 24082042

[3] Deans R Abbott J . Review of intrauterine adhesions. J Minim. Invasive Gynecol. (2010) 17:555–69. doi: 10.1016/j.jmig.2010.04.016 20656564

[4] Yu D Li T-C Xia E Huang X Liu Y Peng X . Factors affecting reproductive outcome of hysteroscopic adhesiolysis for asherman’s syndrome. Fertil. Steril. (2008) 89:715–22. doi: 10.1016/j.fertnstert.2007.03.070 17681324

[5] Johary J Xue M Zhu X Xu D Velu PP . Efficacy of estrogen therapy in patients with intrauterine adhesions: Systematic review. J Minim. Invasive Gynecol. (2014) 21:44–54. doi: 10.1016/j.jmig.2013.07.018 23933351

[6] Roy KK Baruah J Sharma JB Kumar S Kachawa G Singh N . Reproductive outcome following hysteroscopic adhesiolysis in patients with infertility due to asherman’s syndrome. Arch Gynecol. Obstet. (2010) 281:355–61. doi: 10.1007/s00404-009-1117-x 19455349

[7] Guo EJ Chung JPW Poon LCY Li TC . Reproductive outcomes after surgical treatment of asherman syndrome: A systematic review. Best Pract Res Clin Obstet. Gynaecol. (2019) 59:98–114. doi: 10.1016/j.bpobgyn.2018.12.009 30713131

[8] Page MJ McKenzie JE Bossuyt PM Boutron I Hoffmann TC Mulrow CD . The PRISMA 2020 statement: an updated guideline for reporting systematic reviews. BMJ (2021), n71. doi: 10.1136/bmj.n71 33782057 PMC8005924

[9] Jauniaux E Bunce C Grønbeck L Langhoff-Roos J . Prevalence and main outcomes of placenta accreta spectrum: a systematic review and meta-analysis. Am J Obstet. Gynecol. (2019) 221:208–18. doi: 10.1016/j.ajog.2019.01.233 30716286

[10] Silver RM Branch DW . Placenta accreta spectrum. N Engl J Med (2018) 378:1529–36. doi: 10.1056/NEJMcp1709324 29669225

[11] Tikkanen M . Placental abruption: epidemiology, risk factors and consequences: Placental abruption, epidemiology. Acta Obstet. Gynecol. Scand (2011) 90:140–9. doi: 10.1111/j.1600-0412.2010.01030.x 21241259

[12] Rosenberg A . The IUGR newborn. Semin Perinatol. (2008) 32:219–24. doi: 10.1053/j.semperi.2007.11.003 18482625

[13] Peipert JF Donnenfeld AE . Oligohydramnios: a review. Obstetrical gynecological survey (1991) 46(6):325–39. doi: 10.1097/00006254-199106000-00002 2067755

[14] American Diabetes Association . Classification and diagnosis of diabetes: Standards of medical care in diabetes–2019. Diabetes Care (2019) 42:S13–28. doi: 10.2337/dc19-S002 30559228

[15] Garmi G Salim R . Epidemiology, etiology, diagnosis, and management of placenta accreta. Obstet. Gynecol. Int (2012) 2012:1–7. doi: 10.1155/2012/873929 PMC3356715 22645616

[16] Favilli A Tosto V Ceccobelli M Parazzini F Franchi M Bini V . Risk factors for non-adherent retained placenta after vaginal delivery: a systematic review. BMC Pregnancy Childbirth (2021) 21:268. doi: 10.1186/s12884-021-03721-9 33789611 PMC8015016

[17] Liu C-n Yu F Xu Y Li J Guan Z Sun M . Prevalence and risk factors of severe postpartum hemorrhage: a retrospective cohort study. BMC Pregnancy Childbirth (2021) 21:332. doi: 10.1186/s12884-021-03818-1 33902475 PMC8077797

[18] Panelli DM Phillips CH Brady PC . Incidence, diagnosis and management of tubal and nontubal ectopic pregnancies: a review. Fertil. Res Pract (2015) 1:15. doi: 10.1186/s40738-015-0008-z 28620520 PMC5424401

[19] Twesigomwe G Migisha R Agaba DC Owaraganise A Aheisibwe H Tibaijuka L . Prevalence and associated factors of oligohydramnios in pregnancies beyond 36 weeks of gestation at a tertiary hospital in southwestern Uganda. BMC Pregnancy Childbirth (2022) 22:610. doi: 10.1186/s12884-022-04939-x 35918640 PMC9344782

[20] Wang W Xie X Yuan T Wang Y Zhao F Zhou Z . Epidemiological trends of maternal hypertensive disorders of pregnancy at the global, regional, and national levels: a population-based study. BMC Pregnancy Childbirth (2021) 21:364. doi: 10.1186/s12884-021-03809-2 33964896 PMC8106862

[21] Zhou T Du S Sun D Li X Heianza Y Hu G . Prevalence and trends in gestational diabetes mellitus among women in the united states 2006–2017: A population-based study. Front Endocrinol (2022) 13:868094. doi: 10.3389/fendo.2022.868094 PMC9207520 35733768

[22] Romo A Carceller R Tobajas J . Intrauterine growth retardation (IUGR): epidemiology and etiology. Pediatr Endocrinol Rev PER (2009) 6(3):332–6. 19404231

[23] Valle RF Sciarra JJ . Intrauterine adhesions: Hysteroscopic diagnosis, classification, treatment, and reproductive outcome. Am J Obstet. Gynecol. (1988) 158:1459–70. doi: 10.1016/0002-9378(88)90382-1 3381869

[24] Yamamoto N Takeuchi R Izuchi D Yuge N Miyazaki M Yasunaga M . Hysteroscopic adhesiolysis for patients with asherman’s syndrome: menstrual and fertility outcomes. Reprod Med Biol (2013) 12:159–66. doi: 10.1007/s12522-013-0149-x PMC5892977 29662367

[25] Feng Y Li X Xiao J Li W Liu J Zeng X . Relationship between placenta location and resolution of second trimester placenta previa. J Huazhong Univ Sci Technolog. Med Sci (2017) 37:390–4. doi: 10.1007/s11596-017-1745-5 28585139

[26] Pabuçcu R Atay V Orhon E Urman B Ergün A . Hysteroscopic treatment of intrauterine adhesions is safe and effective in the restoration of normal menstruation and fertility. Fertil. Steril. (1997) 68:1141–3. doi: 10.1016/S0015-0282(97)00375-0 9418714

[27] Fernandez H . Operative hysteroscopy for infertility using normal saline solution and a coaxial bipolar electrode: a pilot study. Hum Reprod (2000) 15:1773–5. doi: 10.1093/humrep/15.8.1773 10920101

[28] Salma U Xue M Md Sayed AS Xu D . Efficacy of intrauterine device in the treatment of intrauterine adhesions. BioMed Res Int (2014) 2014:1–15. doi: 10.1155/2014/589296 PMC4165200 25254212

[29] Dawood A Al-Talib A Tulandi T . Predisposing factors and treatment outcome of different stages of intrauterine adhesions. J Obstet. Gynaecol. Can (2010) 32:767–70. doi: 10.1016/S1701-2163(16)34618-7 21050509

[30] Hooker AB Mansvelder FJ Elbers RG Frijmersum Z . Reproductive outcomes in women with mild intrauterine adhesions; a systematic review and meta-analysis. J Matern. Fetal Neonatal Med (2022) 35:6933–41. doi: 10.1080/14767058.2021.1931103 34044740

[31] Committee on Practice Bulletins—Gynecology . ACOG practice bulletin no. 191: Tubal ectopic pregnancy. Obstetrics gynecology (2018) 131(2):e65–77. doi: 10.1097/AOG.0000000000002464 29232273

[32] Gestational hypertension and preeclampsia: ACOG practice bulletin, number 222. Obstetrics gynecology (2020) 135(6):e237–60. doi: 10.1097/AOG.0000000000003891 32443079

[33] Perlman N. C. Carusi D. A . (2019). Retained placenta after vaginal delivery: risk factors and management. International journal of women's health, 11:527–534. doi: 10.2147/IJWH.S218933 PMC6789409 31632157

