# Supplementary figures and images for: A meta-analysis of obstetric and neonatal outcomes in patients after treatment of hysteroscopic adhesiolysis
Source: Front Endocrinol (Lausanne). 2023 Mar 24;14:1126740. doi: 10.3389/fendo.2023.1126740 (PMC10080075; doi:10.3389/fendo.2023.1126740)

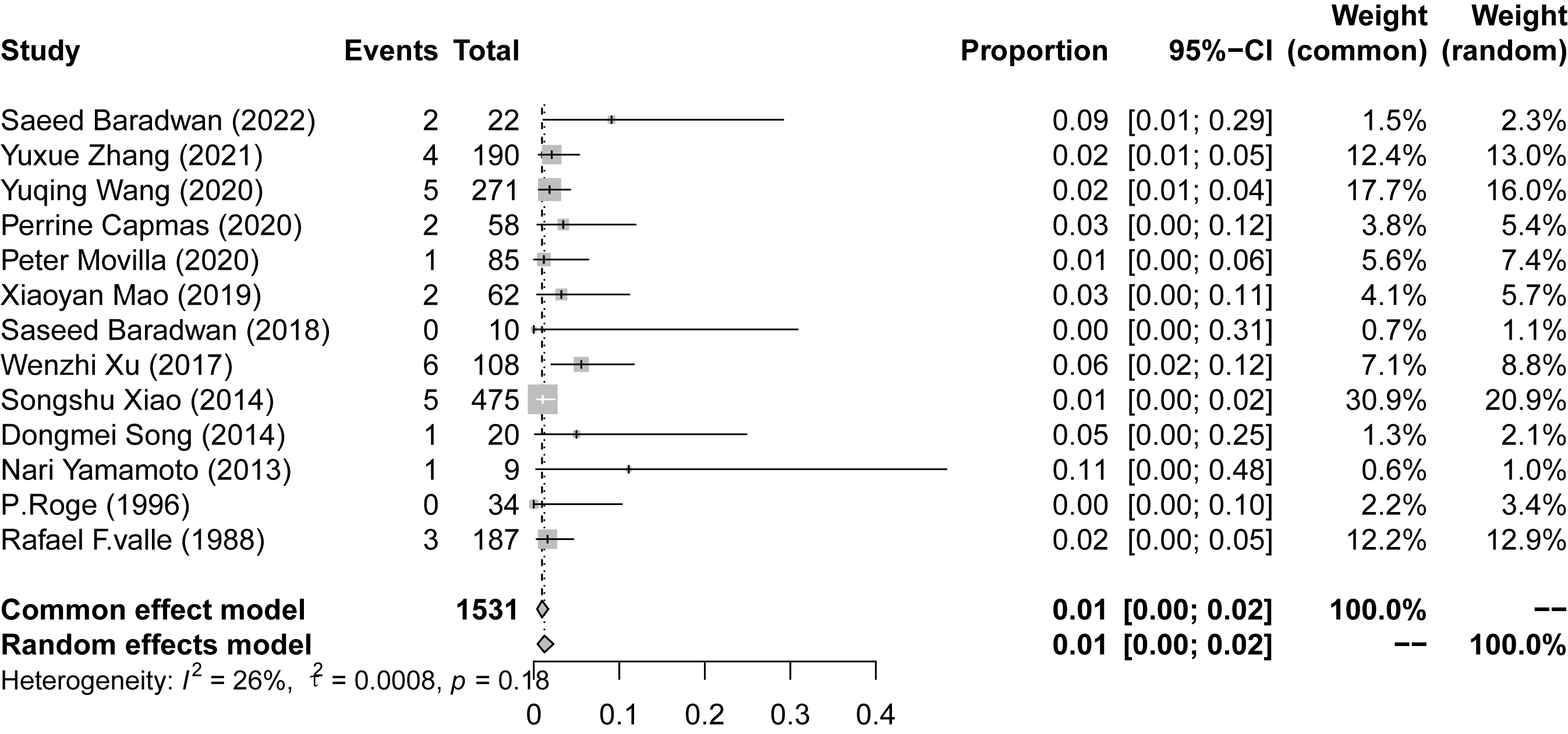

Supplement: Supplementary Figure 1 — Forest plot diagrams of ectopic pregnancy (A), oligohydramnios (B), intrauterine growth restriction (C), gestational hypertension (D), and gestational diabetes mellitus (E). [file Image_1.tif]

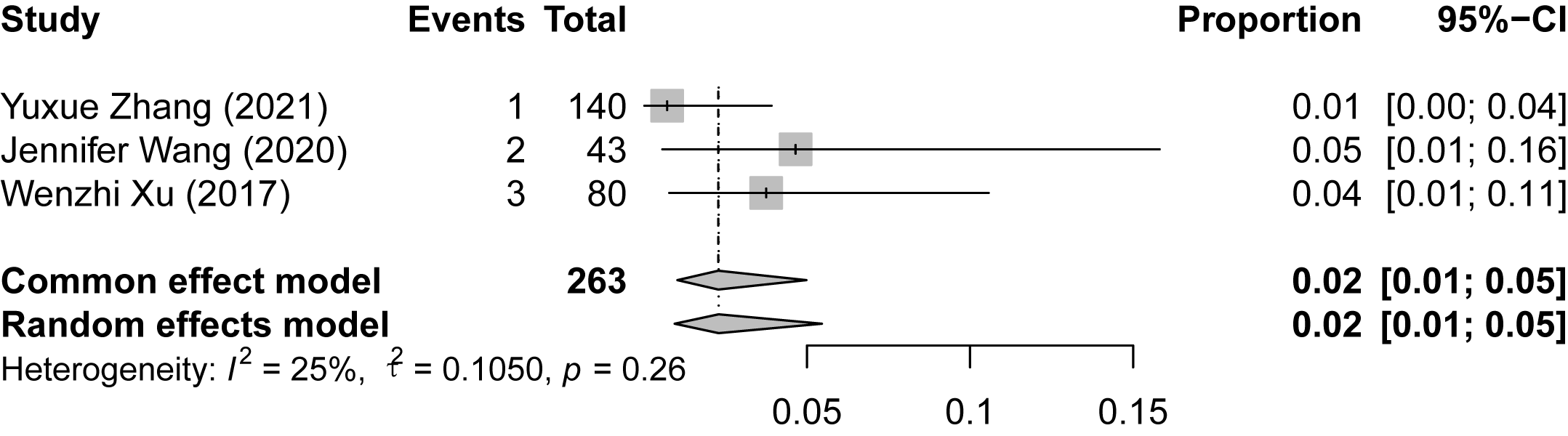

Supplement: Supplementary Figure 2 — The funnel plots of placenta accreta (A), postpartum hemorrhage (B) and ectopic pregnancy (C) which have more than 10 studies reported. [file Image_2.tif]

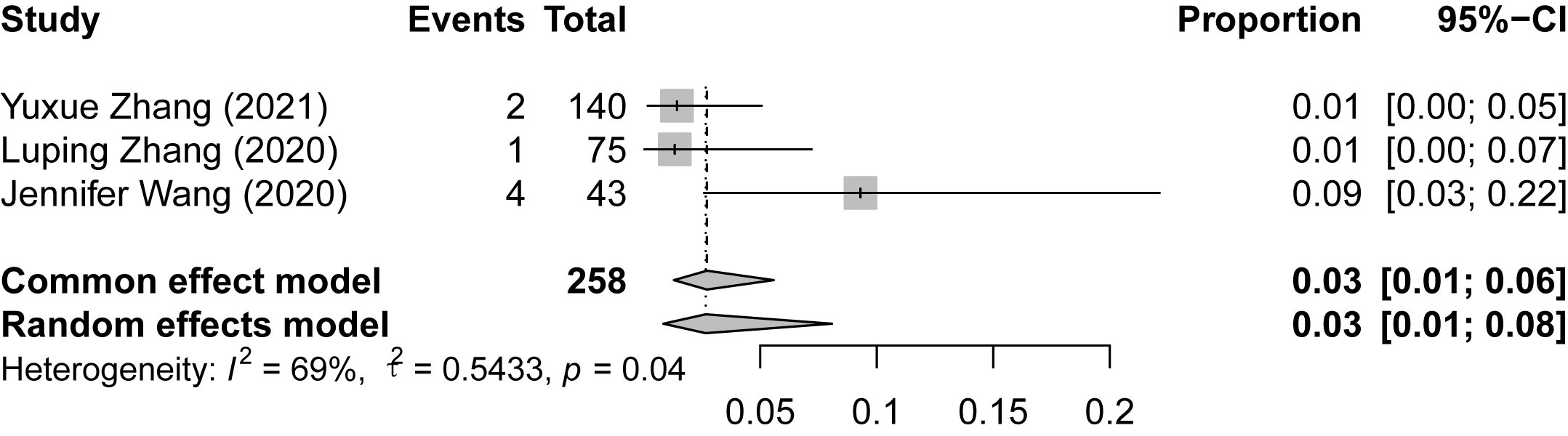

Supplement: Supplementary file 3 [file Image_3.tif]

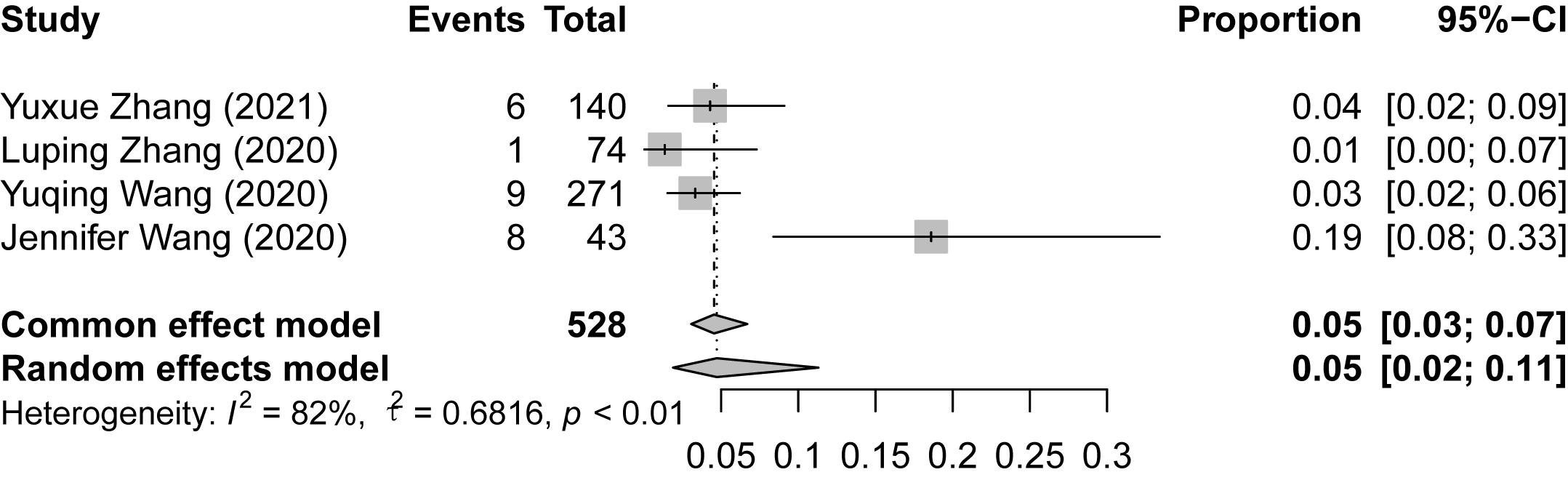

Supplement: Supplementary file 4 [file Image_4.tif]

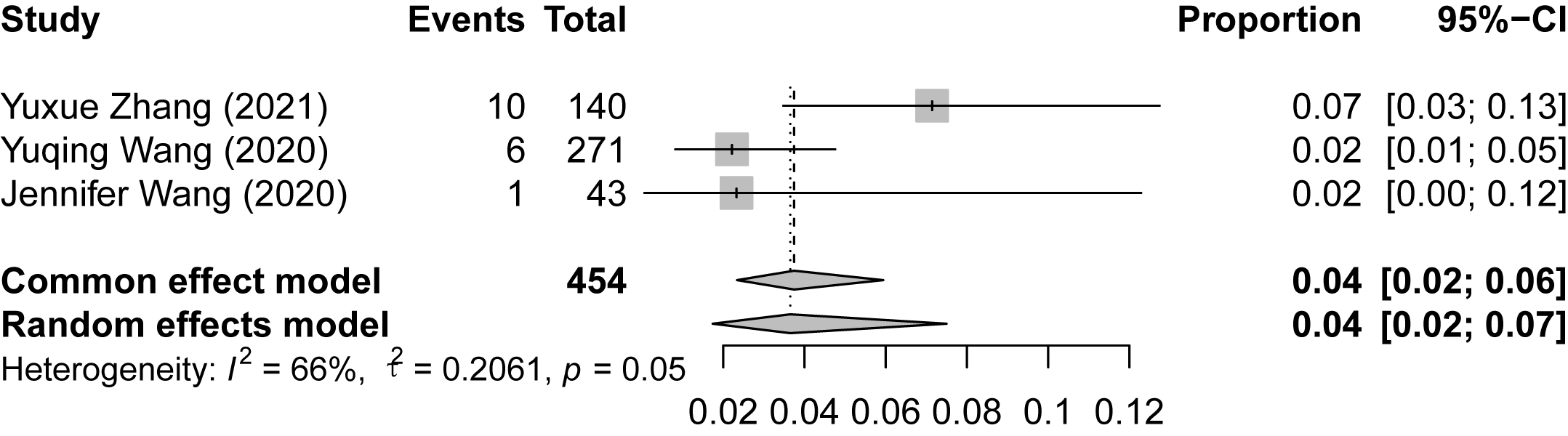

Supplement: Supplementary file 5 [file Image_5.tif]

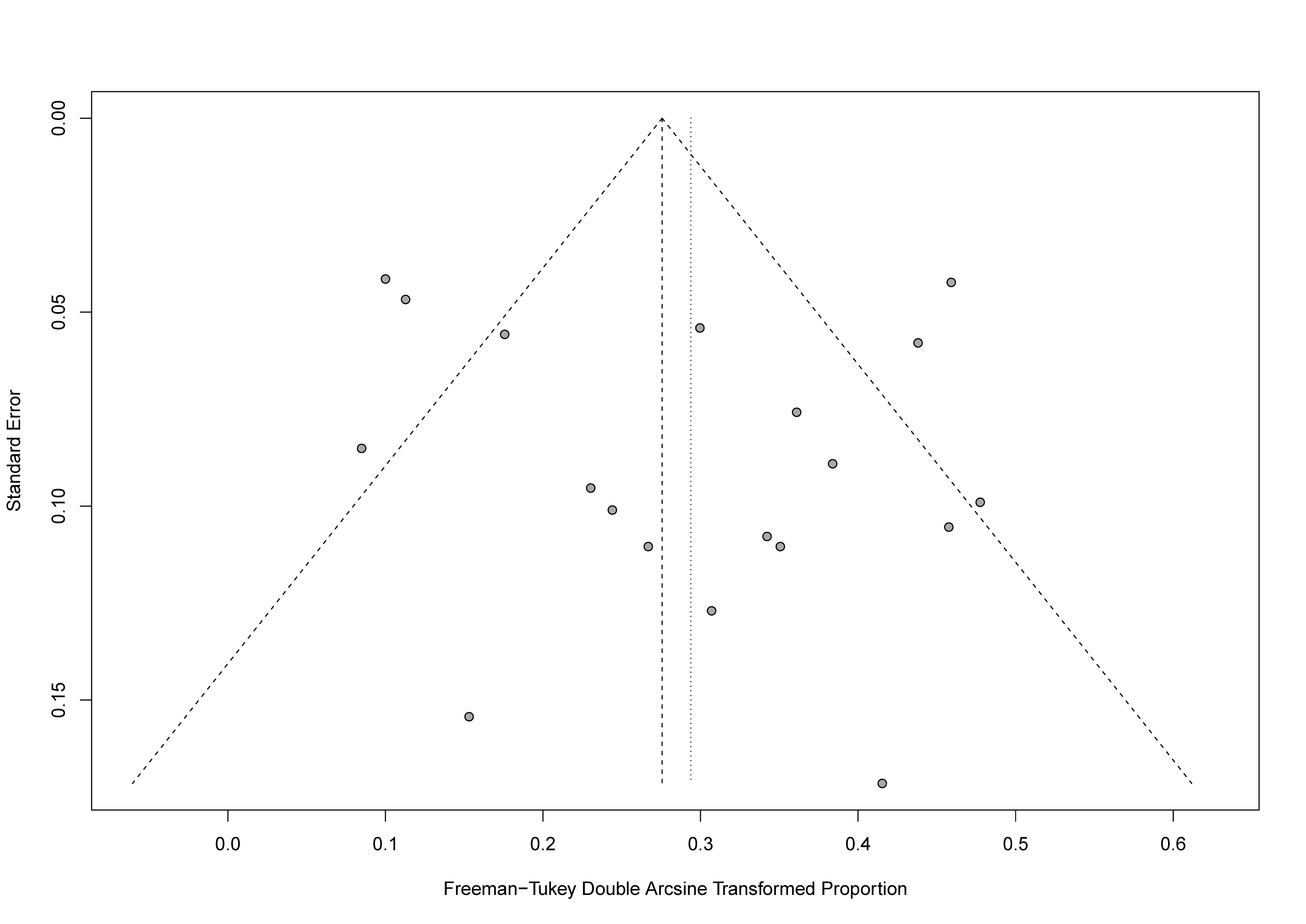

Supplement: Supplementary file 6 [file Image_6.tif]

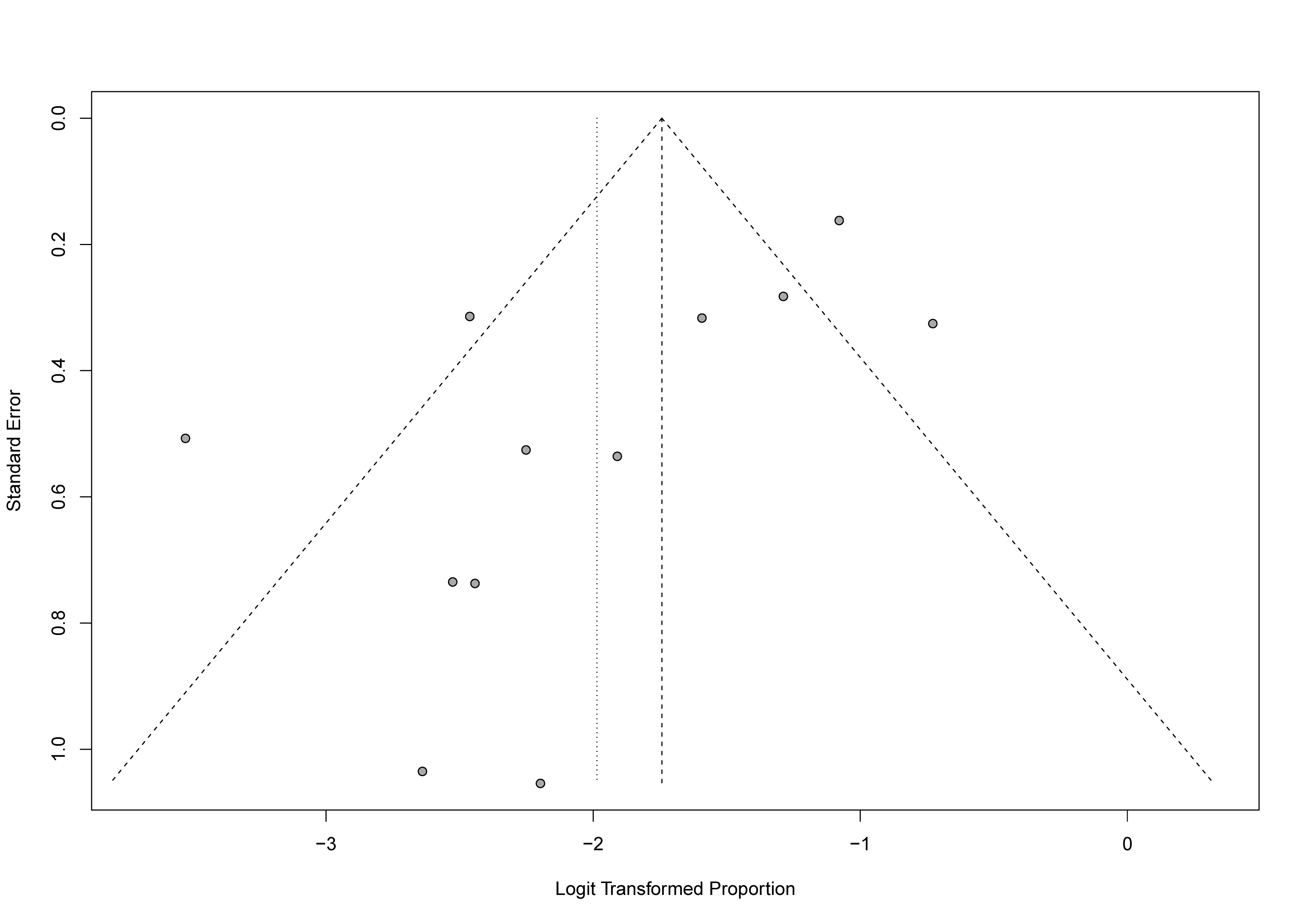

Supplement: Supplementary file 7 [file Image_7.tif]

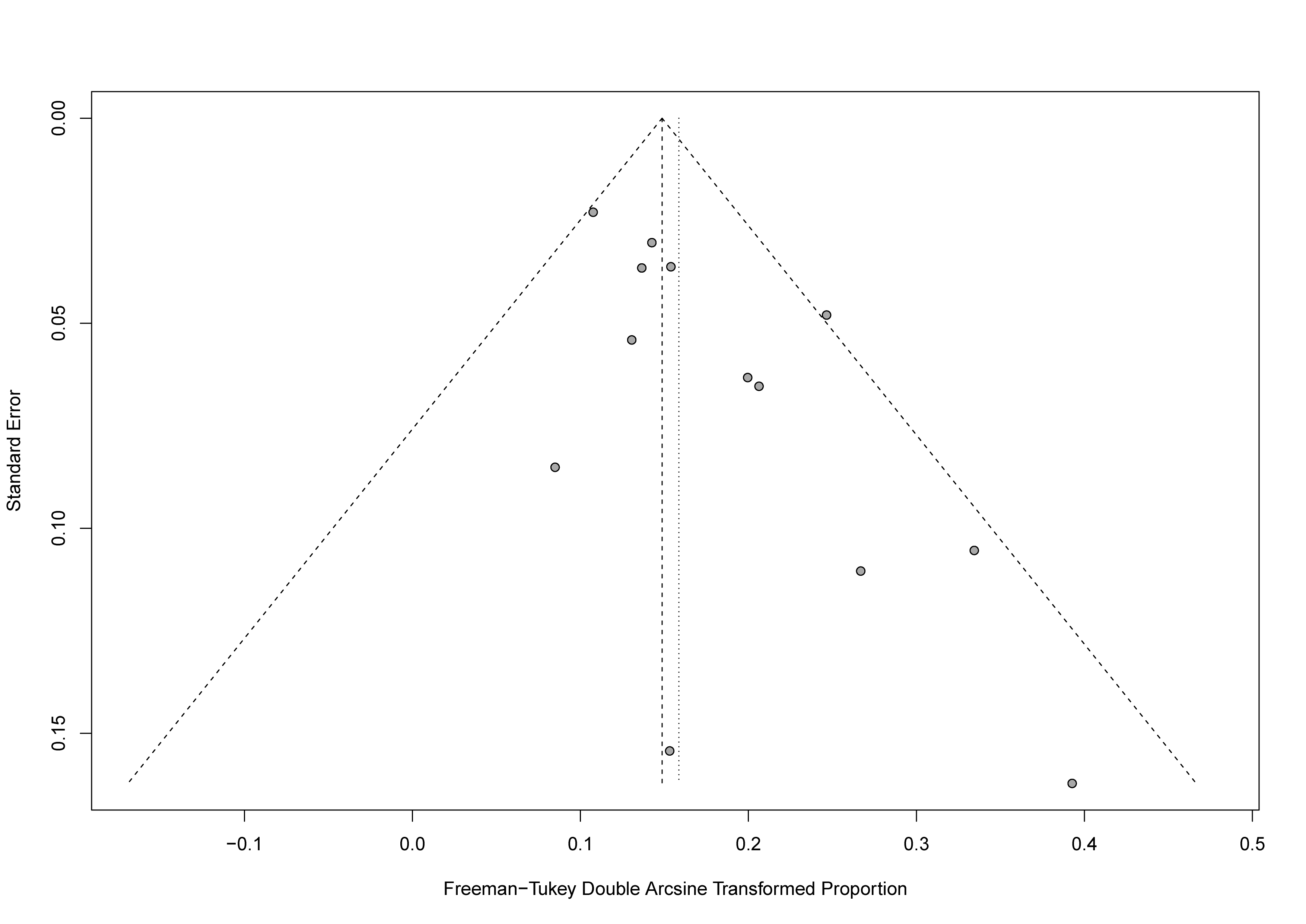

Supplement: Supplementary file 8 [file Image_8.tif]
